# Safety and effectiveness of methylphenidate ER multi-unit pellet system in ADHD patients: An open label study

**DOI:** 10.4102/sajpsychiatry.v30i0.2267

**Published:** 2024-11-14

**Authors:** Renata Schoeman, Evelyn Y. Lai, Anne-Marie Nel, Mashra Gani, Muhammed A. Fulat, Akbar A. Mahomed

**Affiliations:** 1 Stellenbosch Business School, Stellenbosch University, Stellenbosch, South Africa; 2 Department of Medical Affairs, Mylan (Pty) Ltd, Johannesburg, South Africa; 3 Robert Broom Medical Centre, Krugersdorp, South Africa; 4 Smartmed CTC, Port Elizabeth, South Africa; 5 Clinical Trial Systems, Pretoria, South Africa; 6 Zinakekele Medical Centre, Mpumalanga, South Africa

**Keywords:** attention deficit hyperactive disorder, Contramyl XR, effectiveness, methylphenidate, multiple-unit pellet system, treatment experienced, treatment naïve, Weiss Functional Impairment Rating Scale

## Abstract

**Background:**

Attention deficit hyperactive disorder (ADHD) is a neurodevelopmental disorder occurring in children and adults. Pharmacotherapy remains the cornerstone of ADHD treatment. Stimulants such as methylphenidate are effective and have been one of the best studied and most frequently used treatment for ADHD. However, different delivery mechanisms and devices may potentially impact patient experience and real-life outcomes.

**Aim:**

This study evaluated the effectiveness of Multiple-Unit Pellet System Delivered Extended-Release Methylphenidate (Contramyl XR) on symptom control and reported outcomes in ADHD patients, in a real-world setting.

**Setting:**

A phase IV, open label, flexible dose, prospective, observational study conducted at six sites covering five provinces of South Africa.

**Methods:**

About 119 participants with ADHD (both newly diagnosed [treatment-naïve] and methylphenidate-treated [switch-over] patients) were enrolled and initiated either on Contramyl XR or switched over from methylphenidate to Contramyl XR. Primary efficacy was assessed by Weiss Functional Impairment Rating Scale (WFIRS) over 12 weeks.

**Results:**

In all, 117 participants completed the study (treatment-naïve patients: 46% [*n* = 55] and switch-over patients: 54% [*n* = 64]). Mean change from baseline in total WFIRS (95% confidence interval) was –17.7 (–21.1, –14.3; *p* < 0.001) at week 4 and –29.3 (–33.5, –25.2; *p* < 0.001) at week 12. At week 12, there was significant improvement in WFIRS scores, with treatment satisfaction reported by treatment-naïve patients. Switch-over patients also demonstrated comparable effectiveness.

**Conclusion:**

Contramyl XR was found to be clinically effective either as de novo or as switch therapy. It was well tolerated, and all patients chose to continue with the treatment option.

**Contribution:**

Despite distinct and different delivery mechanism of Contramyl XR, this study provides evidence for using it as an alternate treatment option versus reference methylphenidate, in both treatment-naïve and switch-over ADHD patients. Study participants willingness to continue Contramyl XR therapy post study, further strengthens the confidence on the effectiveness of Contramyl XR in managing ADHD patients.

## Introduction

Attention deficit hyperactivity disorder (ADHD) is a neurodevelopmental condition found in both child and adult populations. In Africa, prevalence of ADHD among children and adolescents has been reported to be approximately 5% – 8%,^1^ and up to 70% of individuals still experience clinically significant symptoms in adulthood.^2^ In South Africa, the prevalence of ADHD is similar to the prevalence rates reported in Africa.^3,4^ The ADHD has a profound impact on children’s performance at school, in the work environment for adults and influences the functioning of the family and social environments.

Prior to the treatment initiation, a comprehensive clinical assessment and definite diagnosis are crucial and become the cornerstone of diagnosis. It is NOT a tick box exercise, and a definite diagnosis is NOT made just using the rating scales (which merely act as screening instruments). Not only is a comprehensive family and developmental history crucial but also the collateral information from school, parents or partner are required. Symptoms should be present across the lifespan, and across domain, and meet the Diagnostic and Statistical Manual of Mental Disorders criteria-four or five (DSM-IV or DSM-V) diagnostic criteria for ADHD.

The South African ADHD guidelines recommend multi-disciplinary and multimodal (psychosocial intervention and/or medication regime) treatment approach along with disease management (including treatment compliance) for patients with confirmed ADHD diagnosis and is crucial in preventing complications and the associated long-term costs.^2,5^ Pharmacotherapy remains the main stay of treatment for ADHD. Psychostimulants such as methylphenidate, dextroamphetamine and mixed amphetamine salts are considered as first-line drugs because of their proven safety and effectiveness. Non-psychostimulants including atomoxetine, clonidine, guanfacine are regarded as second-line agents for the treatment of ADHD.^6,7,8^

Methylphenidate is a central nervous system (CNS) stimulant that has been well established as a safe and effective treatment for ADHD since several decades.^9^ It is thought to block the reuptake of norepinephrine and dopamine into the presynaptic neurons and increase the release of these monoamines into the extra-neuronal space. It is a racemic mixture comprised of the d- and l-isomers. The d-isomer is more pharmacologically active than the l-isomer.^10^

Methylphenidate is generally considered to be safe. Most adverse effects are, however, mild and self-limited, and the benefits of the medication for ADHD often outweigh the risks. Common side effects include headache, sleep disturbances, decreased appetite and others. Serious adverse events are uncommon although growth retardation is observed in children when taken long-term.

Contramyl XR is an approved extended-release (ER) methylphenidate and is indicated for the treatment of ADHD in children and adolescents (6–17 years of age), and adults (18–65 years of age), who meet the DSM-IV or DSM-V criteria for ADHD. In comparison to the ER osmotic release oral system (OROS) methylphenidate, Contramyl XR uses multiple-unit pellet system (MUPS) because modified, controlled release system is proven to superimpose the same biphasic release of methylphenidate. Multiple-unit pellet system is one of the two most widely used delivery systems for continuous and controlled release of methylphenidate, the other being OROS. Some of the other ER oral delivery systems of methylphenidate include spheroidal oral drug absorption system (SODAS, e.g. Ritalin LA^®^), modified release pellets (e.g. Medikinet MR^®^), OROS (e.g. Concerta^®^, Neucon^®^, Mefedenil^®^, Unicorn MPH^®^), hydrophilic matrix release system (e.g. Radd^®^) and ER film coated tablets (e.g. Acerta^®^).

Multiple-unit pellet system combines advantages of both tablets and pellet-filled capsules in one dosage form. It offers several advantages including reduced risk of local irritation and toxicity, better predictable bioavailability, reduced probabilities of dose dumping as well as minimal changes in the plasma drug concentrations.^11^ With a limited body of evidence supporting MUPS delivered methylphenidate, this study aimed to evaluate the effectiveness of Contramyl XR in ADHD patients, in a real-world outpatient clinical practice setting. The objectives of this study were to assess the combined improvement in study participants’ clinical response and functionality over 12 weeks of treatment (assessed by the Weiss Functional Impairment Rating Scale [WFIRS], patient-reported or caregiver rated and clinician interpreted), and to determine the correlation between WFIRS and Clinical Global Impressions (CGI) scales based on the changes.

## Research methods and design

This was a phase IV, open label, flexible dose, prospective, observational study to evaluate the safety and effectiveness of MUPS delivered ER methylphenidate in patients with ADHD in a real-world clinical practice setting. This study was conducted at six sites covering the five provinces of South Africa.

### Study population

A total of 119 participants with a confirmed diagnosis of ADHD were included in the study. The study population comprised of children and adolescents (6–17 of years age) and adults (18–65 years of age). Study participants were categorised into two groups – newly diagnosed ADHD patients, with no prior exposure to methylphenidate (treatment naïve patients) and ADHD patients with previous treatment experience or prior exposure to other methylphenidate products and who switched-over to Contramyl XR (switch-over patients). Switch-over patients were taking either short acting methylphenidate, other long-acting medications or other methylphenidate products prior to being prescribed with Contramyl XR.

This study was designed to closely mimic the clinical practice in a real-world setting, wherein, most of the ADHD patients were diagnosed, treated and managed by psychiatrists (adults and paediatrics), paediatricians, general practitioners or family physicians.

Patients were prescribed Contramyl XR as per the approved indications and dosing in the label. Also, for these patients Contramyl XR was prescribed as ‘no substitution’ written on the prescription (i.e. the clinician did not allow the pharmacist to substitute a different generic formulation). Among the treatment naïve patients, children and adolescents received 18 mg methylphenidate once daily while adults received 18 or 36 mg methylphenidate once daily. In case of switch-over patients, who were on methylphenidate thrice daily at doses 15 mg/day – 60 mg/day, dose conversion was recommended based on clinical judgement. Likewise, patients on 5 mg, 10 mg, 15 mg, or 20 mg methylphenidate hydrochloride twice daily or thrice daily, received 18 mg, 36 mg, 54 mg, or 72 mg Contramyl XR once daily, respectively. Dosage was adjusted at weekly intervals, at an increment of 18 mg up to a maximum of 54 mg/day for children, 72 mg/day for adolescents 108 mg/day for adults. The average initial dose of Contramyl XR received in the total study population was 27 mg (range: 18 mg – 54 mg), in treatment naïve patients it was 18 mg and in switch-over patients it was 27 mg.

The clinical decision to commence Contramyl XR was made prior to initiation of any study specific procedures. Potential participants were then approached to participate in this study with written informed consent (or assent form in children) obtained before initiating any screening procedures to confirm the study eligibility.

Prescriptions of Contramyl XR were based on confirmed ADHD diagnosis, which was in line with the licensed prescribing information and patients were enrolled into the study only after all eligibility criteria were met. The decision to prescribe Contramyl XR was based on the clinical appropriateness and also taking into consideration several underlying factors (e.g. refractory and/or existing therapy is intolerable, cost constraints and wanting to switch to cheaper alternatives). Based on its appropriateness, the clinical judgement to prescribe Contramyl XR was at the discretion of treating clinician.

### Inclusion criteria

Males or females with a diagnosis of ADHD according to the DSM-IV or DSM-V or the International Classification of Diseases 10 (ICD-10) criteria were included in this study. Those participants who were literate and were fully able to understand and complete the study-related questionnaires were included. Female patients of childbearing potential and male patients who agreed to use adequate contraception during trials were included in this study.

### Exclusion criteria

Patients who experienced medical conditions that contraindicated the use of methylphenidate were excluded from the study. Patients with a history of or current alcoholism, alcohol abuse, substance abuse or abuse of prescription medication, history of Cannabis use for the last 12 months, familial history of Tourette’s syndrome and impaired liver and renal function were excluded.

Although this was an observational study, it excluded the participants who participated in any ongoing clinical study of any other investigational drug or device within the last 30 days. Female patients with a positive pregnancy test (done at the screening visit) or pregnant or breast-feeding were not included in this study. Participants were advised to avoid alcohol consumption as it may exacerbate the adverse CNS effects of Contramyl XR.

### Study design and endpoints

**FIGURE 1:**
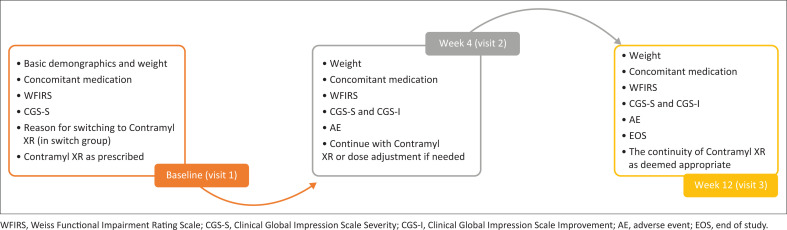
Study design and study endpoints.

A schematic representation of the study design and the study endpoints are shown in Figure 1. The patient demographics such as age, gender, race, height and weight were recorded at the screening or baseline visit. Information on concomitant medications taken 7 days prior to the study and for the duration of the study were recorded at each visit. The study duration was 12 weeks (baseline visit [visit 1, day 1], treatment period [visit 2, week 4 and visit 3, week 12] and end of treatment or early withdrawal visit). The patients or their care givers were administered with WFIRS, CGI-Improvement (CGI-I) and CGI-Severity (CGI-S) scales during each visit, that is, at baseline visit (visit 1, day 1), treatment period (visit 2, week 4 and visit 3, week 12), and end of treatment or early withdrawal visit. The WFIRS was recorded as reported by the patient and/or parent, while CGI-I and CGI-S scales were completed by the treating clinician.

The WFIRS is a validated scale, available in two versions: WFIRS – self-report (WFIRS-S) and WFIRS – parent report (WFIRS-P), that assesses ADHD specific functional impairment on clinically relevant domains. It allows clinicians to determine the improvement in ADHD and patients’ functional difficulties, when used before and after treatment. The WFIRS-S and WFIRS-P consists of 69 items and 50 items, respectively. Both the scales are rated on a 4-point Likert scale and capture the impact of patient’s emotional or behavioural problems in the last month. In both the scales, items have been categorised under several domains such as family, school, work among others, and any item being rated 2 or 3 signifies considerable functional impairment. In this study, for every patient, the total score or mean score of total score or number items for each domain was calculated and as per DSM-IV criteria, any domain with at least two items scored 2, one item scored 3 or a mean score > 1.5 was considered impaired.^12,13^

The CGI-S and CGI-I scales are clinician rated scales that assess the global functioning of a patient before and after starting the medication and severity of illness in the past 7 days based on a 7-point Likert scale. The CGI-S scoring of 1 denotes patient being normal and 7 as most extremely ill, while CGI-I scoring of 1 represents substantial improvement in patients’ symptoms since the start of the treatment and a score of 7 as worse since the initiation of treatment.^14^

During the first visit of the patients, apart from the demographics, rating of the scales, the reason for patients switching from other methylphenidate treatments, the reasons for clinical decision to switch medications were recorded. Subsequently, patients were prescribed the appropriate dose of Contramyl XR (once daily as per prescribing information) and were reviewed during visit 3 (4 weeks ± 5 days). In addition to recording the weight, WFIRS, CGI-I and CGI-S scales, the patients were also reviewed for dose adjustments during each visit (visits 1, 2 and 3). Symptom improvement reported by the clinicians and the functional improvement by patients were evaluated.

Contramyl XR (with dose optimisation) was prescribed for 3 months duration while participants were in the study. Once participants had completed the study, the clinician managed them according to the clinician’s standard of care and were re-evaluated as appropriate to their individual needs.

The primary endpoint is the composite score of WFIRS (patient-reported and/or parent or caregiver rated) at week 4 and week 12 compared with baseline. The secondary endpoints included the CGI-I and CGI-S scores (clinicians reported and/or rated) on the participant’s level of functionality and symptoms improvement at week 4 and week 12 compared with baseline and as well as correlation of the WFIRS and CGI at week 4 and week 12.

Safety and tolerability were also assessed by review of adverse event reporting. Adverse events, if any, and their causal relationship to Contramyl XR were reported and attributed by the clinical investigator. Adverse events were elicited by open ended questions such as ‘how have you been feeling since you started taking medication’ or ‘have you noticed any changes since you started taking medication’. Patients received the appropriate doses of Contramyl XR and were assessed (as per the week 4 visit) at 12 weeks (± 5 days; visit 3 = end of study visit [EOS]). If participants withdrew early from the study or stopped medication, assessments were made (as per the EOS visit) at the time of withdrawal.

### Statistical analysis

#### Sample size calculation

The study sample size was calculated as 50 participants in each group with a two-sided 95% confidence interval (CI) for the change from baseline WFIRS (primary endpoint). For WFIRS to be considered statistically significant, the mean change from baseline was –4 with the 95% CI falling within the range of –5.66 to –2.34 for a study sample size of 50. The mean of 95% CI was based on calculated extent of 1.663 from the observed mean. Therefore, the 95% CI for the mean of –4 will be falling within –5.66 to –2.34.

Based on the sample size calculation, this study planned for a sample size of 150 participants, with a minimum of 50 participants per study group and six study sites within South Africa.

Descriptive statistics were applied for qualitative and quantitative data. The mean total WFIRS, CGI-I and CGI-S were calculated at visits 1, 2 and 3, and the mean differences from visit 1 till visit 3 were computed at 95% CI. This mean difference was also compared in subgroup analyses for participants aged > 18 years and < 18 years and in previously diagnosed (switch over patients) and treatment naïve patients. Mean and standard deviation were computed for continuous variables. Counts and percentages were computed for categorical variables.

The primary endpoints of changes from baseline in the total scores of WFIRS versus WFIRS at visits 2 and 3 were analysed, and these analyses were conducted in both treatment naïve and switch-over patients, among both children and/or adolescents (< 18 years) and adult patients. A mean change of -4 from baseline score of WFIRS with 95% CI falling within the range of –5.66, –2.34 was predetermined to be statistically significant. Secondary endpoints were analysed to assess the correlation between the changes from baseline in WFIRS versus CGI-S, baseline WFIRS versus CGI-I at visits 1, 2 and 3 using the Pearson correlation coefficients and *p*-values < 0.05 were considered to be statistically significant.

### Ethical considerations

Ethical approval to conduct this study was obtained from Pharma-Ethics Independent Research Ethics Committee (No. 130622600).

## Results

A total of 119 participants were enrolled in this study. Of these, 117 participants completed the 12-week treatment period. Two participants were withdrawn from the study as they had taken Contramyl XR prior to the participation in the study. Despite their early withdrawal from the study, they were included in the analysis of the outcomes and data recorded at the last visit when they were seen (EOS) were used in the analysis (i.e. last observation carried forward). Patients were actively recruited from six sites, which included Krugersdorp and Pretoria (Gauteng), Western Cape, KwaZulu-Natal, Eastern Cape and Mpumalanga in South Africa.

The baseline demographic and clinical characteristics of the study participants are presented in Table 1. Among the total study population, 55% (*n* = 65) of the patients belonged to > 18 years of age. The average age of the participants was 18 ± 1.05 (range: 15–62 years). Male (68%; *n* = 81) participants were more compared to females. With respect to race, black people (34%; *n* = 41) were more compared to white participants (28%; *n* = 33). The mean height of the participants was 1.6 metres (range: 1.0 m – 1.9 m) and mean weight was 51.4 kg (range: 16 kg – 114 kg).

**TABLE 1:** Baseline demographic and clinical characteristics (*N* = 119).

Characteristics	Number of patients	%
**Age (Years)**		
> 18	65	55
< 18	54	45
**Gender**		
Male	81	68
Female	38	32
**Race**		
Black people	41	34
White people	33	28
Indian people	30	25
Other	15	13
Newly diagnosed patients	55	46
Switch-over patients	64	54

The average initial dose of Contramyl XR was 27 mg (range: 18 mg – 54 mg) in the total study population, 18 mg in treatment naïve patients and 27 mg in switch-over patients. The average initial dose of Contramyl XR in patients < 18 years of age and in those > 18 years of age was 18 mg and 27 mg, respectively.

Among the switch-over patients, 56% (*n* = 36) of them switched over to Contramyl XR because of a lack of effect with previous treatment and 30% (*n* = 19) of them because of financial constraints. Twenty-four participants cited ‘Other reasons’ for switch-over, however, in 21 patients this was because of a lack of effect with the previous treatment.

Patients who switched-over to Contramyl XR were previously on medications such as amitriptyline (23.1%; *n* = 15), methylphenidate ER tablets (23.1%; *n* = 15) or capsules (13.8%; *n* = 9), and methylphenidate 10 mg tablets (30.8%; *n* = 20).

The most common reasons for the patients in switch over group to get started on Contramyl XR included financial constraint (29.7%; *n* = 19), inadequately controlled ADHD symptoms from the existing methylphenidate treatment (23.4%; *n* = 15), possible intolerable side effect from existing therapy (4.7%; *n* = 3) and others (37.5%; *n* = 24) (Table 2).

**TABLE 2:** Reasons for switching to Contramyl XR (*N* = 64).

Reasons	Number of patients	%
Financial constraint	19	29.7
Possible intolerable side effect from existing therapy	3	4.7
Uncontrolled from existing methylphenidate	18	28.1
Wanting to switch from immediate release/short acting methylphenidate to extended-release option	3	4.7
Others†	21	32.8

† , Reasons include: ADHD symptoms despite amitriptyline treatment (*n* = 1), because of small difference (*n* = 1), ineffective duration (*n* = 1), minimal change in ADHD symptoms (*n* = 5), no significant change in ADHD symptoms (*n* = 3), parent reluctant to initiate with methylphenidate (*n* = 2), patient has turned 6 years and can use a stimulant now (*n* = 1), patient requires improvement (*n* = 1), poor control (*n* = 1), symptoms of ADHD still present, no significant changes in symptoms of ADHD (*n* = 2), treatment was not effective (*n* = 1), not medicated enough as per patient’s mother (*n* = 1), poor improvement with current regimen (*n* = 1).

In all the study participants treated with Contramyl XR, the mean change in the total WFIRS was statistically significant at visits 2 and 3 when compared with baseline visit. Also, study participants showed significant improvement in ADHD symptom control and daily functioning. The improvement in ADHD symptoms were identified and correlated to both CGI-S and CGI-I at visits 2 and 3, among the various patient subgroups evaluated (Table 3).

**TABLE 3:** A summary statistics for total Weiss Functional Impairment Rating Scale, Clinical Global Impression Scale Severity and Clinical Global Impression Scale Improvement.

Study outcome measures	Number of patients	Mean ± s.e.	95% CI
**Total WFIRS and WFIRS changes from baseline**			
WFIRS visit 1	119	63.9 ± 3.6	56.7 to 71.1
WFIRS visit 2	119	46.2 ± 3.2	39.9 to 52.6
WFIRS visit 3	118	35.0 ± 2.4	30.3 to 39.7
WFIRS change from baseline Visit 2	119	−17.7 ± 1.7	−21.1 to −14.3
WFIRS change from baseline Visit 3	118	−29.3 ± 2.1	−33.5 to −25.2
**Newly diagnosed participants**			
WFIRS change from baseline Visit 2	55	−19.3 ± 2.5	−24.4 to −14.3
WFIRS change from baseline Visit 3	54	−33.3 ± 2.1	−38.7 to −27.9
**Switch participants**			
WFIRS change from baseline Visit 2	64	−16.3 ± 2.3	−20.9 to −11.7
WFIRS change from baseline Visit 3	64	−26.0 ± 3.1	−32.2 to −19.9
**Under 18 years (children and adolescents)**			
WFIRS change from baseline Visit 2	66	−13.8 ± 2.0	−17.7 to −9.9
WFIRS change from baseline Visit 3	65	−19.2 ± 2.2	−23.6 to −14.8
**Adult participants**			
WFIRS change from baseline Visit 2	53	−22.5 ± 2.8	−28.2 to −16.8
WFIRS change from baseline Visit 3	53	−41.8 ± 3.0	−47.8 to −35.7
**CGI scales and changes from baseline**			
CGI-S visit 1	119	3.3 ± 0.1	3.1 to 3.6
CGI-S visit 2	119	2.5 ± 0.1	2.3 to 2.7
CGI-S visit 3	118	2.1 ± 0.1	2.0 to 2.3
CGI-S change from baseline Visit 2	119	−0.8 ± 0.1	−1.0 to −0.7
CGI-S change from baseline Visit 3	118	−1.2 ± 0.1	−1.4 to −0.9
CGI-I Visit 2	119	2.2 ± 0.1	2.1 to 2.4
CGI-I Visit 3	118	1.9 ± 0.1	1.8 to 2.1

CI, confidence interval; s.e., standard error; WFIRS, Weiss Functional Impairment Rating Scale; CGI, Clinical Global Impressions; CGI-S, Clinical Global Impressions-Severity of illness scale; CGI-I, Clinical Global Impressions-Global Improvement scale.

**FIGURE 2:**
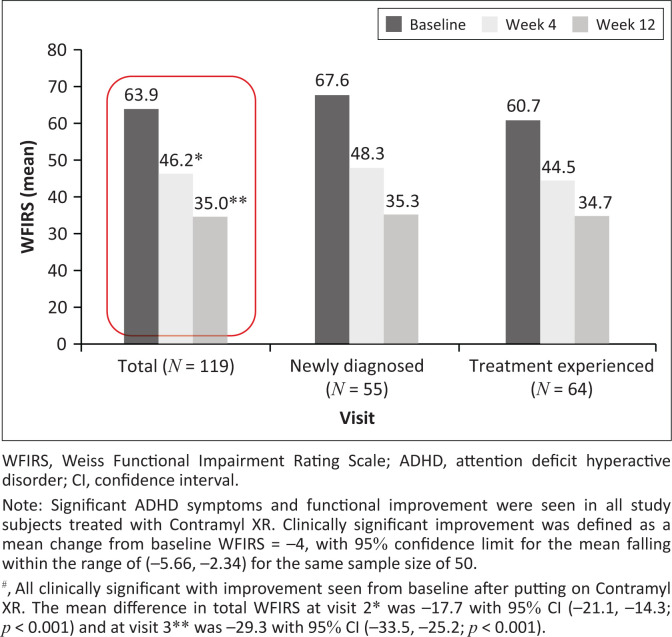
Mean change in total Weiss Functional Impairment Rating Scale from baseline^#^ at each visit.

**FIGURE 3:**
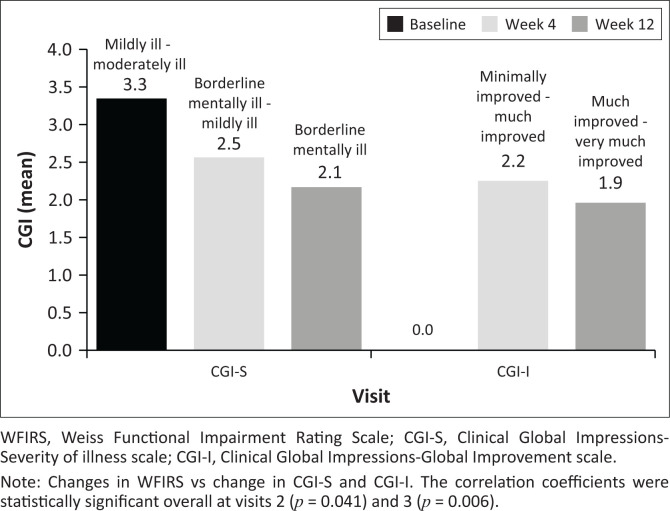
Mean change in total Clinical Global Impression Scale Severity and Clinical Global Impression Scale Improvement from baseline versus total Weiss Functional Impairment Rating Scale at each visit.

Figure 2 and Figure 3 show the summary statistics for total WFIRS and CGI-S, and for changes from baseline at visits 1, 2 and 3. In the switch-over group, the total number of participants who were previously diagnosed and on treatment was 65. Of these 65 participants, one participant received methylphenidate ER tablets and methylphenidate long-acting capsules at the same time prior to study enrolment. Hence, the total count of previously treated participants was considered as 64.

For changes in WFIRS versus CGI-S, the correlation coefficients were statistically significant overall at visits 2 (*p* = 0.041) and 3 (*p* = 0.006) (Figure 3). Also, in newly diagnosed patients, correlation coefficient showed that the changes in total WFIRS versus CGI-S and total WFIRS versus CGI-I at visit 2 was statistically significant (*p* < 0.001). At visit 3, similar statistical significance was observed with total WFIRS versus CGI-S but not for change in total WFIRS versus CGI-I (Figure 3).

Even in the previously diagnosed patients, the change in total WFIRS versus CGI-S and total WFIRS versus CGI-I at visits 2 and 3 were statistically significant (*p* < 0.001). However, no statistical significance was observed in the change in total WFIRS versus CGI-S in these patients at visit 2.

**FIGURE 4:**
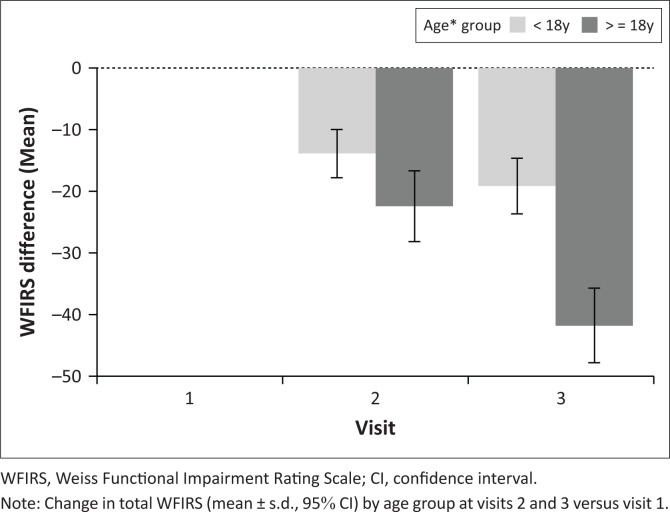
Change in Weiss Functional Impairment Rating Scale from baseline in participants < 18 years versus > 18 years.

The subgroups of patients, both < 18 years of age and > 18 years of age, showed significant change in the total WFIRS versus CGI-I (*p* < 0.05) at visit 2 only (Figure 4).

The mean weight of the study participants at visits 1, 2 and 3 were 51.4 kg, 51.3 kg and 51.7 kg, respectively. Compared with visit 1, the mean difference (95% CI) in weight at visits 2 and 3 were –0.1 kg (–0.3, 0.1) and +0.3 kg (0.0, 0.7), respectively. This small but statistically significant difference at visit 3 was observed in all subgroups studied. For switch-over patients, the mean difference (95% CI) at visit 3 was +0.7 kg (0.2, 1.2). For patients < 18 years of age, the mean difference (95% CI) was +0.4 kg (0.0, 0.8).

A total of 12 adverse events (AEs) were reported during the study period. Three participants reported headache (2.5%), two reported insomnia (1.7%), two reported irritability (1.7% including one manifested in the late afternoon). Other AEs reported included appetite loss, going quiet, spaced out, dry mouth, anxiety. All AEs were mild to moderate in severity, with majority (83%) assessed as possibly or probably associated with Contramyl XR. Most of the patients reporting AEs recovered or resolved without sequelae (67%), while information on resolution for rest of the participants was still ongoing or undocumented.

## Discussion

The main objective of this study was to provide more data in supporting the safety and effectiveness of MUPS ER methylphenidate, as extensive data on the role of OROS methylphenidate in the treatment of ADHD is available.

Multiple-unit pellet system methylphenidate has been proven to be bioequivalent to the biphasic OROS methylphenidate based on the pharmacokinetic and pharmacodynamic studies. However, there is a paucity of data or paucity of head-to-head comparisons, which can demonstrate the effectiveness of MUPS methylphenidate especially in switch over patients who previously were exposed to other methylphenidate formulations including OROS controlled release formulation.

This study was a phase IV study, conducted by clinicians in their day-to-day clinical work in managing patients diagnosed with ADHD. Although many phase III studies have confirmed the efficacy of methylphenidate in ADHD in clinical research,^15,16^ this study has expanded the body of knowledge on the effectiveness of methylphenidate as well as the impact of switching to generic alternatives in ADHD patients that clinicians encounter during their daily practice.

This study provides the evidence supporting the use of MUPS as an alternative treatment option in patients diagnosed with ADHD, demonstrated by improvements in ADHD symptoms and daily functioning of the patients in a real-world setting, irrespective of whether the patient was treatment-naïve or switched over from previous ADHD treatment, and its safety and effectiveness were seen in both children or adolescents and in adults.

The study met its objectives, namely demonstrating that effectiveness by change in WFIRS from baseline to 12 weeks was both statistically and clinically relevant in both patients who were newly diagnosed in addition to those who required a switch in treatment (this is true even in those participants where financial consideration is the main reason for switching). These changes were equally relevant in both children and/or adolescents (< 18 years of age) and adults (> 18 years of age). Correlations showed no clinically relevant differences between treatment groups or age groups. In this study, patients were started at the lowest effective dose of the stimulant and based on patients’ need the dose was increased or titrated considering factors such as their age, weight, among others. The impact of medication on weight gain or growth in children and adolescents < 18 years of age was not seen, although the study period was limited to 3 months.

The correlated changes, interpreted as improvement in functionality and severity of illness, were statistically significant for visits 2 and 3 when compared to baseline prior initiation of Contramyl XR. Reports of adverse events were low, mild, transient, and none resulted in participants withdrawing from the study or from treatment.

The data further support treating clinician’s confidence in offering MUPS as an alternate treatment option, especially in circumstances of financial constraint as one of the considerations when making treatment choices and methylphenidate is a preferred ADHD therapy. However, further studies are required to see if a similar impact can be achieved in patients switching over from OROS methylphenidate to Contramyl XR.

## Conclusion

Contramyl XR is clinically effective in those patients who were using it as initial therapy as well as in those who substituted it with switch therapy. This study further supports the interchangeability of Contramyl XR as a generic formulation to its reference methylphenidate, including other long-acting formulations, and switching is well received by patients at all age groups.

### Limitations

One of the limitations of this study is the small patient population. Also, the number of patients in the switch-over study group was small, as the number of patients who were previously exposed to other methylphenidate formulations were limited.

In this study, the study period was 12 weeks. Although no impact of the medication on weight gain or growth in children and adolescents < 18 years of age was seen, the short study period is a limitation for a meaningful conclusion to be drawn.

The prospect of substance abuse because of the use of stimulants and the potential impact on patients in a real-life scenario was not considered in this study.
